# Case report: Novel variants cause developmental and epileptic encephalopathy in three unrelated families from Mali

**DOI:** 10.3389/fgene.2024.1412442

**Published:** 2024-11-18

**Authors:** Salia Bamba, Lala Sidibé, Seybou H. Diallo, Lassana Cissé, Kékouta Dembélé, Abdoulaye Yalcouyé, Weizhen Ji, Mohamed Emile Dembélé, Salimata Diarra, Alassane dit Baneye Maiga, Oumou Traoré, Salimata Diallo, Samuel Ephrata Mefoung, Amadou Touré, Adama Koné, Lauren Jeffries, Cheick O. Guinto, Emily K. Mis, Kenneth H. Fischbeck, Mustafa K. Khokha, Saquib A. Lakhani, Guida Landouré

**Affiliations:** ^1^ Faculté de Médecine et d’Odontostomatologie, University of Sciences Techniques and Technologies of Bamako, Bamako, Mali; ^2^ Pediatric Genomics Discovery Program (PGDP), Department of Pediatrics, Yale University School of Medicine, New Haven, CT, United States; ^3^ Service de Pédiatrie, Centre Hospitalier Universitaire de Gabriel Touré, Bamako, Mali; ^4^ Service de Neurologie, Centre Hospitalier Universitaire de Gabriel Touré, Bamako, Mali; ^5^ Hôpital du District de la Commune IV, Bamako, Mali; ^6^ Clinique Khaïdara, Bamako, Mali; ^7^ Service de Neurologie, Centre Hospitalier Universitaire du Point G, Bamako, Mali; ^8^ Neurogenetics Branch, NINDS, NIH, Bethesda, MD, United States

**Keywords:** developmental epileptic encephalopathies (DEEs), exome sequencing, novel variant, sub-Saharan, Mali

## Abstract

**Background and Objectives:**

Developmental and epileptic encephalopathies (DEEs) are a group of neurological disorders characterized by early-onset seizures that are often resistant to treatment, by electroencephalographic abnormalities, and by developmental delay or regression. Their genetic basis remains largely unelucidated, especially in sub-Saharan Africa (SSA). We investigated the genetic bases of DEE in three Malian families.

**Methods:**

Patients underwent clinical evaluation, and DNA was obtained for whole exome sequencing (WES). Putative variants were screened in all available family members and *in silico* prediction analyses were performed to assess pathogenicity.

**Results:**

Five patients from three unrelated families with DEEs had symptoms that started during the neonatal period with seizures and myoclonus that became refractory to antiepileptic medications. WES identified previously unreported variants in all three families: homozygous variants in *GRIN1* and *SYNJ1,* and compound heterozygous variants in *RARS2*. These variants affected protein structure by *in silico* tools and were classified as variants of uncertain significance hot, pathogenic/likely pathogenic respectively according to ACMG criteria.

**Discussion:**

We identified rare variants in three genes (*GRIN1, SYNJ1,* and *RARS2*) associated with early onset of DEEs in SSA, expanding their genetic and epidemiological spectrum. Larger cohort studies in SSA may unravel more variants with potential clinical implications and further our understanding of the disease mechanism.

## Introduction

Developmental and epileptic encephalopathies (DEEs) are a group of neurological disorders characterized by early-onset seizures that are often resistant to treatment, by electroencephalographic abnormalities, and by developmental delay or regression (Happ and Carvill, 2020). Previously thought to be caused by acquired factors, the discovery of monogenic mutations through next-generation sequencing has revealed a genetic basis for some DEE subtypes (McTague et al., 2016). While the genetic etiology of DEEs is increasingly recognized worldwide, only a few cases have been genetically diagnosed in sub-Saharan Africa (SSA) due to limited access to genetic facilities (Esterhuizen et al., 2018). In this study, we report novel variants causing DEEs in the Malian population.

## Methods

### Standard protocol approvals, registrations, and patient consents

This study was in compliance with the declaration of Helsinki and ethics approval was obtained from the Faculté de Médecine et d’Otondostomalogie, Université des Sciences, des Techniques et des Technologies de Bamako (N°2020/129/CE/FMOS/FAPH). Written informed consent/assent was obtained from all participants and/or legal guardians.

### Clinical and laboratory assessment

Patients were examined by neurologists, pediatricians, and medical geneticists. Blood chemistries, brain imaging, and electroencephalography (EEG) were performed in selected available patients to rule out acquired causes and refine phenotypic descriptions.

### Genetic analysis

DNA was extracted from peripheral blood using the Puregene Blood DNA kit C (Qiagen, Germantown, MD) following the manufacturer’s instructions. WES was performed in trios where possible for each family. Variant calling, annotation, and prioritization as well as prediction for deleteriousness are detailed in Supplementary Material S1. Segregation of candidate variants in available family members was done by Sanger sequencing. Variants were classified according to American College of Medical Genetics (ACMG) criteria (Richards et al., 2015).

### Molecular modeling

Protein sequences of relevant protein domains for *GRIN1* (NP_000823.4), *SYNJ1* (NP_001153774.1) and RARS2 (NP_001337434.1) were obtained from the National Center of Biotechnology Information (NCBI). Three-dimensional (3D) structures of mutant proteins were modelled on SWISS-MODEL server and newly predicted structures were refined on Galaxy Web server (https://galaxy.seoklab.org/). Pymol served for structure visualization and hydrogen bonds analysis.

## Results

Clinical and genetic findings are summarized in Table 1.

**TABLE 1:** Phenotypic and genetic findings in patients with DEE.

Patients	Clinical examination findings										Laboratory findings	
	Age (Y)	Sex	Age onset (D)	First symptom	Tremor	Seizure types	Developmental delay	Hypotonia	Motor weakness	Visual loss	EEG	Variant
F1.IV-2	25 D	M	4	Seizures	None	Myoclonic, spasms	Yes	None	None	None	Not done	*GRIN1: NM_007327.4:*c.1703T > C; p.Leu568Pro, Homozygous
F2.IV-1	7	F	10	Seizures	None	BCS, focal motor seizures	Yes	Yes	Yes	None	Abnormal	*SYNJ1:* NM_203446.3*:*c.1255C > T; p.Arg380*, Homozygous
F2.IV-2	2	M	2	Seizures	None	BCS, focal motor seizures	Yes	Yes	Yes	None	Not done	
F3.III-1	3	M	2	Convulsive seizures	Yes	Clonic Seizures	Yes	Yes	None	Yes	Abnormal	*RARS2:* NM_020320.5*:* c.422A > G; p.His141Arg and c.449T > C; p.Ile150Thr Compound Heterozygous
F3.III-2	1	M	3	Convulsive seizures	Yes	Clonic Seizures	Yes	Yes	None	Yes	Abnormal	

F: family; Y: years; Day: D; BCS: bilateral clonic seizures.

**FIGURE 1:**
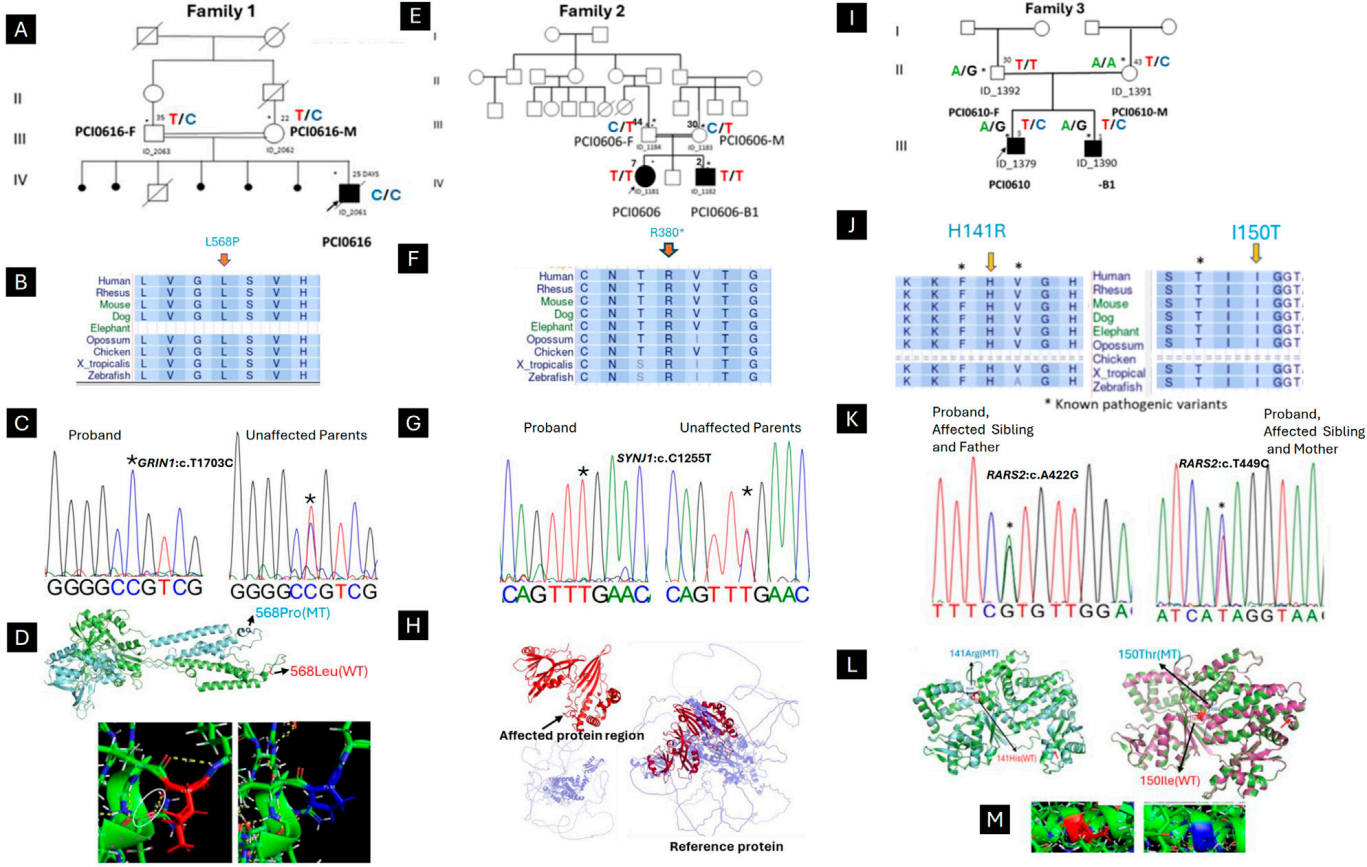
Clinical and genetic findings of DEE patients (Family 1,2,3). **(A)** Pedigree of the case with neonatal myoclonic epilepsy showing recessive inheritance pattern (arrow indicates the proband and asterisks the participants seen in clinic). **(B)** The horizontal rectangle at the bottom represents the amino acid alignment around the mutated residue and their corresponding conservation among selected species using the UCSC Genome Browser. **(C)** Chromatogram showing the c.1703T > C variant in *GRIN1*. **(D)** Superimposed structure of GRIN1 wild type (green) and mutant (light blue), showing the shift of 3D conformational between. Hydrogen bond analysis showing the loss of bonding interaction of wild type leucine (white arrow) with phenylalanine in the mutant. **(E)** Pedigree of the case with infantile epileptic encephalopathy showing recessive inheritance pattern (arrow indicates the proband and asterisks the participants seen in clinic). **(F)** The horizontal rectangle at the bottom represents the amino acid alignment around the mutated residue and their corresponding conservation among selected species using the UCSC Genome Browser. **(G)** Chromatogram showing the **(C)** 1138C > T variant in *SYNJ1.* **(H)** Structure of the SYNJ1 reference protein, with the affected region highlighted in red. The truncated protein (in red) and the remaining portion of the reference protein (in light blue) illustrate the consequences of a premature stop codon, leading to the rupture of the full-length structure and the loss of both the α-helix and β-sheet regions. **(I)** Pedigree of the case with Ponto-cerebellar hypoplasia with seizures and showing recessive inheritance pattern (arrow indicates the proband and asterisks the participants seen in clinic). **(J)** The horizontal rectangle at the bottom represents the amino acid alignment around the mutated residue and their corresponding conservation among selected species using the UCSC Genome Browser.). **(K)** Chromatogram showing the compound heterozygous in *RARS2* with both affected children (c.422A > G inherited from Father and c.449T > C inherited from Mother). **(L)** Superimposed structure of wild type RARS2 (green) and mutant (p.His141Arg; light blue) showing the partial loss of helix in the mutant (red arrow). **(M)** Superimposed structure of wild type RARS2 (magenta) and mutant (p.Ile150Thr; green) showing the partial loss of helix in the mutant (red arrow). Hydrogen bond analysis showing that there is not significant difference in bonding interaction between Isoleucine-150 and Threonine-150.

Family 1 (Figure 1A): A 25-days-old male from a consanguineous marriage and Soninké ethnicity, born after normal pregnancy and delivery, was referred for uncontrolled neonatal seizures and developmental delay. Symptoms started 4 days after birth with myoclonic seizures, spasms in flexion of the upper limbs, and tonic seizures involving the four limbs. Mother reported five miscarriages and premature death of another one-year-old child. WES identified a novel homozygous missense variant in *GRIN1* (NM_007327.4: c.1703T > C; p. (Leu568Pro)) in the proband, categorized as “hot” variant of uncertain significance (VUS-Hot) by ACMG criteria (PM1,PP3, PM2). The Leu568 residue is conserved across a wide range of species (Figure 1B) and the variant is segregating with the disease in the family (Figure 1C). The variant was predicted to disrupt protein 3D conformations and folding, including the loss of four hydrogen bonds between the native Leu589 and Phe591 (Figure 1D). The patient was started on intravenous Sodium Valproate and Clonazepam with no effect and died shortly afterwards.

**FIGURE 2:**
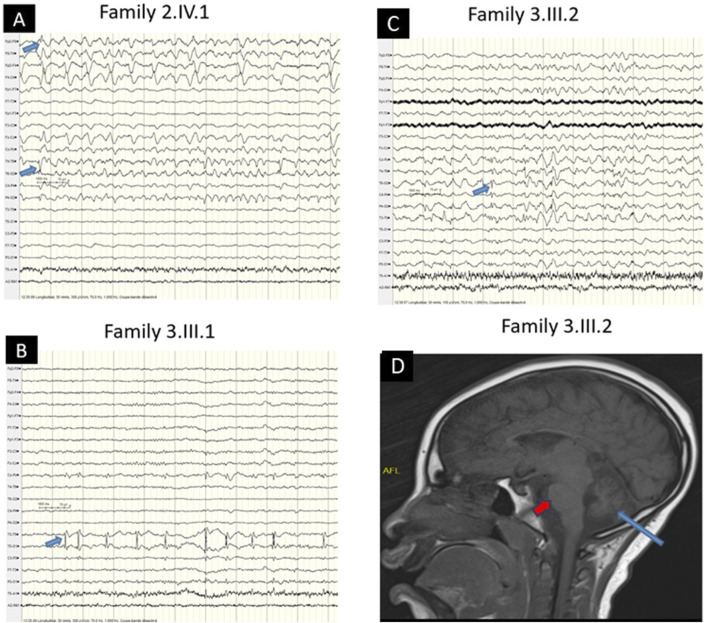
EEG and MRI Characteristics **(A)** EEG showing right temporal spike waves with left low-voltage background activity (blue arrow) in the older patient (Family 2, IV.1). **(B)** EEG showing left temporal spike waves (blue arrow) in the patient (Family 3, III.1). **(C)** EEG showing focal (right temporoparietal) spike waves (blue arrow) in the patient (Family 3, III.2). **(D)** Brain MRI showing cerebellar atrophy (blue arrow) sparing the pons (red arrow) (Family 3, III.2).

Family 2 (Figure 1E): A 7-year-old female proband and her 2-year-old brother from healthy consanguineous parents of Songhai ethnicity, were referred for seizures and motor acquisition delay. Parents reported that around 1 week after birth both patients began having 4–10 bilateral clonic seizures daily. The proband showed delayed motor acquisition, hypotonia with axial weakness, generalized muscle atrophy in the four limbs, multidirectional nystagmus with bilateral Babinski sign, and scoliosis. EEG recorded when the proband was 7 years old, revealed right temporal spike waves and significantly slowed background activity, along with low voltage in the left hemisphere (Figure 2A). Her brother also had motor acquisition delay with generalized hypotonia with inability to sit, muscle atrophy in shoulders and lower limbs, and lumbar hyperlordosis. He had absent tendon reflexes in the four limbs, Babinski sign on the left and multidirectional nystagmus on vertical gaze. WES identified a novel homozygous nonsense variant in *SYNJ1* (NM_203446.3: c.1138C > T; p. (Arg380*)) in both children, classified as likely pathogenic (ACMG criteria PVS1, PM2). The Arg380 residue is conserved across a wide range of species (Figure 1F) and the variant is segregating with the disease in the family (Figure 1G). The premature stop codon, leading to disrupture of the full-length structure of SYNJ1 reference protein and the loss helical structure in the Sac domain and β-sheet regions (Figure 1H). Despite treatment with multiple medications including Carbamazepine, Sodium Valproate, and Clonazepam, both patients continue to experience frequent seizures.

Family 3 (Figure 1I): Male siblings, one and 3 years old, from non-consanguineous healthy parents of Mandingo ethnicity were seen for seizures, psychomotor delay, and visual loss. Symptoms started with tonic clonic seizures within 1 month after birth, and both patients had psychomotor acquisition delay, visual loss, and clonic seizures. Tendon reflexes were brisk in the lower limbs and normal in the upper limbs. In addition, the older patient had a hyperactivity disorder. Sleeping EEG showed left temporal predominant spike waves in the older brother (Figure 2B) and focal right temporoparietal paroxysmal spike waves in the younger (Figure 2C). Brain magnetic resonance imaging of the older child showed cerebellar atrophy sparing the pons (Figure 2D). WES revealed novel compound heterozygous variants in *RARS2* (NM_020320.5: c.422A > G; p. (His141Arg), and c.449T > C; p. (Ile150Thr)). The His141 and Ile150 residues are conserved across a wide range of species (Figure 1J). The p. (His141Arg) and p. (Ile150Thr) variants have been classified as likely pathogenic according to ACMG criteria, with p. (His141Arg) meeting the PP3, PM1, PP5, and PM2 criteria, and p. (Ile150Thr) by PP3, PP5, PM2, and PM3 criteria, respectively. The first variant is inherited from the father and second is inherited from the mother (Figure 1K). Molecular modeling revealed major predicted changes including the loss of helical structures (Figures 1L,M). The patients were being treated with multiple medications including Sodium Valproate, Clonazepam, and Piracetam. However, the treatment was unsuccessful, and the patients continue to experience frequent seizures.

## Discussion

The global prevalence of epilepsy is higher in SSA as compared to other regions of the world (Paul et al., 2012). Although this is partially attributable to factors such as infection and malnutrition, limited access to DNA sequencing undermines the ability to delineate genetic causes (Esterhuizen et al., 2023). Still, the rapid evolvement and decreasing costs of sequencing technologies has facilitated the identification epilepsy genes in SSA, including for DEEs (Esterhuizen et al., 2023). In a recent South African study, genetic variants were detected in 51 of 234 children with DEEs, with *SCN1A* being the most frequently implicated gene (Hebbar and Mefford, 2020). Besides this study, however, reports of molecularly diagnosed DEEs are scarce in SSA. Our work is the first study in Mali examining the genetics of DEEs. We identified unrelated families with DEEs caused by novel pathogenic variants in previously reported genes, *GRIN1*, *SYNJ1* and *RARS2*.

The *GRIN1* gene plays a pivotal role in the proper functioning of N-methyl-D-aspartate (NMDAR) receptor, essential for brain synaptic mechanisms. It is linked to a spectrum of neurological disorders, ranging from DEE to neurodevelopmental disorders, with or without seizures and hyperkinetic movements, showcasing its significant variability in phenotypic expression (Lemke et al., 2016a). An intriguing case from Morocco highlighted a novel mutation associated with intellectual challenges and autism-like features, underlining the gene’s broad impact (Blakes et al., 2022). Our study adds to this with a case from the Sub-Saharan Africa population, emphasizing a rare but classic developmental epileptic encephalopathy phenotype. The p. (Leu568Pro) variant meets the PP3 criterion is supported by the pathogenicity prediction score of 0.911 for human nonsynonymous SNVs (nsSNVs) using the MetaRNN model, which falls between 0.841 and 0.939, indicating that the variant is of moderate pathogenicity. Additionally, the variant satisfies the PM1 criterion, as many missense variants cluster in the transmembrane region close to the mutated Leu568 residue. Therefore, this variant is classified as VUS-Hot, suggesting a probable pathogenic role by the consistent genotype-phenotype correlation and the recessive inheritance pattern observed in the pedigree, which shows severe features associated with a biallelic variant, like the p. Gln556* case (Lemke et al., 2016b), and by the absence of other plausible point or copy number variants in genes associated with DEEs.

The *SYNJ1* gene’s product is key in synaptic vesicle dynamics, with mutations known to lead to early-onset Parkinson’s disease or DEE53, based on the affected protein domain (Hardies et al., 2016). This paper reports on a DEE53 case, characterized by intractable epilepsy and developmental delay, linked to a critical domain mutation (Sac-domain). The p. Arg380* variant is classified as “likely pathogenic” because it satisfies ACMG criteria PVS1 and PM2 based on the fact that it is a null variant with potential loss-of-function mechanism and on its absence in variome databases including gnomAD.

*RARS2* encodes for the mitochondrial arginine-tRNA synthetase, with specific mutations causing pontocerebellar hypoplasia type 6 (PCH6) (Lühl et al., 2016). Clinical spectrum of *RARS2* mutations typically include neurological symptoms such as encephalopathy with intractable seizures and severe developmental delay, primarily affecting the brain. Other organ systems, such as the cardiac, ocular, renal, or hepatic systems, are not commonly involved in this disease (Edvardson et al., 2007). The phenotype varies between the presence or absence of pontocerebellar hypoplasia (PCH). This study describes patients without PCH. While these cases are generally considered to have a milder phenotype, with developmental milestones being relatively normal up to around 6 months, (Nishri et al., 2016) in this study, the two patients contrast with the typical clinical manifestations starting with early infantile psychomotor delay (Lühl et al., 2016). Additionally, the patients have had an unusually prolonged lifespan compared to other affected patients. The phenotypic differences could be due to potential genetic modifiers or be stochastic. According to the ACMG criteria, the first variant in *RARS2* meets the PM1 criterion (moderate) because it is located in the Aminoacyl-tRNA Synthetase Domain of the RARS2 protein, specifically in a short sequence motif HIGH region which has seven reported missense or in-frame deletion variants including three pathogenic or likely pathogenic and four VUS. UniProt (2024).

In summary, we have identified rare genetic variants in *GRIN1, SYNJ1,* and *RARS2* associated with early onset of DEEs in a SSA population from Mali. Our results expand the genetic and epidemiological spectrum of this disease. Larger cohort studies, particularly in other understudied populations, may unravel additional variants that could have implications for their populations and be important in furthering knowledge of the disease mechanism.

## Data Availability

The datasets presented in this article are not readily available because of ethical and privacy restrictions. Requests to access the datasets should be directed to the corresponding author/s.
