# Supplementary material for: Case report: Novel variants cause developmental and epileptic encephalopathy in three unrelated families from Mali
Source: Front Genet. 2024 Nov 18;15:1412442. doi: 10.3389/fgene.2024.1412442 (PMC11609193; doi:10.3389/fgene.2024.1412442)
Supplement: Supplementary file 2 [file Table1.docx]

**Table I**: Phenotypic and genetic findings in patients with DEE

| Patients | *Clinical examination findings* | | | | | | | | |  | *Laboratory findings* | |
| --- | --- | --- | --- | --- | --- | --- | --- | --- | --- | --- | --- | --- |
|  | **Age (Y)** | **Sex** | **Age onset (D)** | **First symptom** | **Tremor** | **Seizure types** | **Developmental delay** | **Hypotonia** | **Motor Weakness** | **Visual Loss** | **EEG** | **Variant** |
| F1.IV-2 | 25 D | M | 4 | Seizures | None | Myoclonic, spasms | Yes | None | None | None | Not done | *GRIN1:*  *NM_007327.4:*c.1703T>C; p.Leu568Pro, Homozygous |
| F2.IV-1 | 7 | F | 10 | Seizures | None | BCS, focal motor seizures | Yes | Yes | Yes | None | Abnormal | *SYNJ1:* *[NM_203446.3](http://www.ncbi.nlm.nih.gov/nuccore/NM_203446.3) :*c.1255C>T; p.Arg380*, Homozygous |
| F2.IV-2 | 2 | M | 2 | Seizures | None | BCS, focal motor seizures | Yes | Yes | Yes | None | Not done |  |
| F3.III-1 | 3 | M | 2 | Convulsive seizures | Yes | Clonic Seizures | Yes | Yes | None | Yes | Abnormal | *RARS2:* *[NM_020320.5](http://www.ncbi.nlm.nih.gov/nuccore/NM_020320.5):* c.422A>G; p.His141Arg and c.449T>C; p.Ile150Thr  Compound Heterozygous |
| F3.III-2 | 1 | M | 3 | Convulsive seizures | Yes | Clonic Seizures | Yes | Yes | None | Yes | Abnormal |  |

F: family; Y: years; Day: D; BCS: bilateral clonic seizures
