# Supplementary material for: Case report: Novel variants cause developmental and epileptic encephalopathy in three unrelated families from Mali
Source: Front Genet. 2024 Nov 18;15:1412442. doi: 10.3389/fgene.2024.1412442 (PMC11609193; doi:10.3389/fgene.2024.1412442)
Supplement: Supplementary file 3 [file Table2.docx]

**Genetic analysis**

**Whole-Exome Sequencing**

ES was performed in trios where possible for each family and putative variants were confirmed by Sanger sequencing. Exomes were captured using IDT xGen exome target capture kit, providing > 96% coverage of RefSeq coding bases with at least 20 independent reads. Enriched libraries were sequenced (2x100) on Illumina HiSeq 4000 instruments with an average of 6 gigabases per exome, giving 50x coverage at 90% of reads. The Varscan.v2.3.9.jar tools were used for variants calling^6^ and annotation was done using ANNOVAR(Wang, K., Li, M., & Hakonarson, H. (2010)) to include information regarding the gene, chromosomal coordinate(s), variants, type of mutation (frameshift, nonsense, nonsynonymous, splicing, and synonymous)Sixteen different predictions of the variant from multiple algorithms, allele frequencies in different databases including gnomAD, Exome Sequencing Project, dbSNP, 1000 Genomes, Complete Genomics, Exome Aggregation Consortium, and annotation of variants in clinical mutation database(ClinVar.2020) were used. Considering the variants annotated, we used the R program to prioritize candidate variants based on the inheritance pattern, annotation, population frequency and gene expression information. We finally considered pathogenic variants on the gene that fit with the family disease classified according to the guidelines of the American College of Medical Genetics (ACMGv3.1). The variants identified were then analyzed using bioinformatics tools to predict their potential impact on the protein structure and function such as Combined Annotation-Dependent Depletion (CADD v1.6)
